# Evolutionary medicine and bioastronautics: an innovative approach in addressing adverse mental health effects to astronauts during long term space missions

**DOI:** 10.3389/fphys.2025.1558625

**Published:** 2025-04-24

**Authors:** Arthur Saniotis, Maciej Henneberg, Kazhaleh Mohammadi

**Affiliations:** ^1^ Department of Medical Microbiology, Cihan University-Erbil, Erbil, Iraq; ^2^ School of Biomedicine, Faculty of Health and Medical Sciences, The University of Adelaide, Adelaide, SA, Australia; ^3^ Institute of Evolutionary Medicine, University of Zurich, Zurich, Switzerland; ^4^ Department of Medical Microbiology, College of Science, Knowledge University, Erbil, Iraq

**Keywords:** evolutionary mismatch, hormonal dysregulation, spaceflight-associated neuro-ocular syndrome, spaceflight-associated dysbiosis, circadian disruption, Earth-out-of-view-phenomenon, relaxed natural selection, evolutionary based countermeasures

## Abstract

Although evolutionary medicine has produced several novel insights for explaining prevalent health issues, it has yet to sufficiently address possible adverse mental health effects of humans during long-term space missions While evolutionary applications to medicine have increased over the past 20 years, there is scope for the integration of evolutionary applications in the new branch of space medicine called bioastronautics, which analyses the effects on human bodies when in outer space. Evolutionary principles may explain what kinds of space environments increase mental health risks to astronauts, both in the short and long term; secondly, evolutionary principles may provide a more informed understanding of the evolutionary mismatch between terrestrial and space environments in which astronauts exist. This information may assist in developing frameworks for improving mental health of astronauts and future space colonists. Consequently, this paper will focus on some of the major evolutionary mismatches currently confronting astronauts’ mental health, with an aim to improve medical knowledge. It will also provide possible therapeutic countermeasures based on evolutionary principles for reducing adverse mental effects on astronauts.

## 1 Introduction

Evolutionary medicine is a growing discipline integrating the principles of evolutionary biology, medicine, genetics and psychology, providing a range of concepts and insights for explaining human vulnerability to disease. (Beeson and Kosal, 2023; Nesse and Williams, 2012; Stearns et al., 2010; Stearns, 2012; Dolgova, 2021). Evolutionary medicine speculates that the human body has evolved under various conflicts of evolutionary forces, trade-offs, mismatches and constraints intrinsic to biological organisms under the influence of natural selection and non-directional forces of evolution such as the genetic drift, gene flow and inbreeding (Nesse and Williams, 2012; Dolgova, 2021). An understanding of these evolutionary phenomena provides a feasible framework for classifying and understanding the complex interaction between genes, environment and history in relation to disease (Dolgova, 2021). Next, the human genome has undergone various adaptations over evolutionary history in response to ancestral environments, disease load and cultural behaviours, which inform current susceptibility to disease (Perry, 2021). Notwithstanding its growth in the English speaking world and in Europe, evolutionary medicine has yet to be established within global medical curricula (Natterson-Horowitz et al., 2023).

Even though evolution has been traditionally considered integral to biology, medical practitioners and public health experts have generally overlooked evolution’s role in human susceptibility to disease. Recent medical science developments such as the Human Genome Project, recombinant DNA, and the Haplotype Map have reaffirmed the significance of genes for human health and disease. Concomitantly, evolutionary psychology examines the gene/environmental interplay and its enduring effect on traits of extant humans.

Thus far, evolutionary medicine has produced several novel insights for explaining prevalent health issues such as cancer, antimicrobial resistance, autoimmune disorders, diabetes, heart disease, depression/anxiety disorders, dementia and the advent of new anatomical variations. Evolutionary medicine also addresses the role of medical and public health interventions in reducing natural selection, which has been proposed to have led to an increase in fitness-reducing alleles with subsequential genetic load (Saniotis et al., 2021; Saniotis et al., 2024). For example, recent studies of 190 countries have suggested a correlation between type-1 diabetes and cancer incidence and the proliferation of harmful genes due to relaxed natural selection (You and Henneberg, 2016; 2017). Alterations in gene patterns due to relaxed selection could be important in mapping out possible future trajectories in human population health.

However, evolutionary medicine has yet to sufficiently address possible adverse mental health effects of humans during long-term space missions (LTSM). “Long term” can be referred to as being in outer space for a couple of months to years (Criscuolo et al., 2020). It will also apply eventually to life in human colonies on celestial bodies (Moon, Mars, *etc.*). While evolutionary applications to medicine have increased over the past 20 years, there is scope for the integration of evolutionary applications in the new branch of space medicine called bioastronautics, which analyses the effects on human bodies when in outer space (Arone et al., 2021). For example, evolutionary principles may explain what kinds of space environments increase mental health risks to astronauts, both in the short and long term; secondly, evolutionary principles may provide a more informed understanding of the evolutionary mismatch between terrestrial and space environments in which astronauts transit. This information may assist in developing frameworks for improving human mental health for future space colonists (Saniotis et al., 2016).

Consequently, this paper will focus on some of the major evolutionary mismatches currently confronting astronauts’ mental health, with an aim to improve bioastronautical knowledge. It will also provide possible therapeutic countermeasures based on evolutionary principles for reducing adverse mental effects to astronauts.

## 2 Evolutionary mismatch, adaptation and outer space

A central concept in evolutionary medicine is “evolutionary mismatch,” which refers to the discordance between the ancestral environments in which hominins lived and the novel societies’ extant human habitat (Trevathan, 2007), resulting from cultural evolution outpacing biological evolution. This is consistent with the rapid rate of allele substitution during the Holocene Epoch, which is thought to be 100 times higher than for most of the evolution of *Homo* (Hawks et al., 2007; Keller, 2008).

It has been speculated that accelerated cultural evolution has left extant humans susceptible to a plethora of diseases and disorders. Like other organisms, the human genetic profile has been shaped by the complex interplay of various evolutionary forces (i.e., natural selection, mutation, gene flow, genetic drift) and sexual selection.

Notwithstanding the evolutionary discordance between ancestral and modern environments, human evolution is on-going, even though, natural selection has been relaxed in our species (Saniotis and Henneberg, 2011b; 2023). Some studies have highlighted rapid human micro-evolution during the Holocene (10,000 ka-present) (Rühli and Henneberg, 2013). Recent microevolution has occurred *via* lactase persistence (Itan et al., 2009), amylase gene copy variation [Zuk, 2013; Perry et al., 2007) malarial resistance, high altitude adaptation, brain volume reduction (Henneberg, 1988; Henneberg and Steyn, 1993)) decreasing musculoskeletal robusticity (Ruff, 2002), and ongoing Abnormal Spindle-like, Microcephaly-associated (ASPM) gene adaptation [Mekel-Bobrov et al., 2005).

Humans have not evolved to live in the outer space environment. This is evident by the numerous studies on the Soviet Mir space station (1986–2001), and American Shuttle and ISS missions, which have shown that even short-term space flight may cause deleterious changes to muscles and skeletal function (Fitts et al., 2001; Adams et al., 2003), vestibular system (Ross and Tomko, 1998), cardiopulmonary system, immunological system, motor system (Kozlovskaya et al., 1981), neuro-behavioural and cognitive function (Kanas and Manzey, 2008; De La Torre et al., 2012).

While understanding of the effect of space flight on the human body and mind has improved in the last two decades, there is still a great deal of medical research needed on mid (1–5 years) to long duration (>5 years) consequences of space flight. The proposed permanent Moon base will provide a basis for monitoring the human body in conditions beyond low Earth orbit. Furthermore, NASA’s intention to send a manned space mission to Mars in the next-generation (the return trip will take approximately 21 months) will further identify health risks due to high exposure to radiation (astronauts could be exposed to ∼1 Sievert (Sv) radiation during the trip) (Cucinotta and Durante, 2006; Wilson et al., 1995), as well as potential neuro-behavioural issues due to the extended duration of the mission.

Evolutionary history indicates that humans exhibit significant plasticity and adaptability across various environments. In the context of evolution, outer space represents a distinct environment that necessitates a range of new adaptations. A key focus is how humans adjust to ensure neuro-behavioural allostasis, which involves sustaining neurohormonal balance for prolonged survival in space. This paper will further explore mental health challenges in bioastronautics, considering the implications of evolutionary mismatch.

## 3 Stress and glucocorticoid dysregulation in outer space

A major area of space medicine is the role of stress during long-duration space missions. Stress has been shown to affect astronaut behaviours and their ability to perform multiple tasks (Kanas, 1985; 1987). Space flight stressors include microgravity, living in small and enclosed confines, isolation, arduous workload, boredom, disconnection from the natural world, circadian disruption, unpredictable events, *etc.* These issues have received various levels of attention from space researchers.

Human stress response (HSR) is mediated by the Hypothalamus-Pituitary-Adrenal axis (HPA). The HPA axis evolved in our pre-hominin ancestors as an adaptive mechanism for triggering the sympathetic nervous system (SNS) in response to threats (de Kloet et al., 2005). Second, the HSR evolved to deal mainly with acute stressors (a few hours) and not with sub-acute (several days) or chronic stress (de Kloet, 2004). Prolonged exposure to chronic stress (several weeks/months to years) has been shown to play a role in depressive disorder and psychotic illness (de Kloet, 2004). In the hippocampus, long term glucocorticoid hormonal insults lead to impaired declarative and spatial memory [Ortiz and Conrad 2018), with diminished fear learning. Although dendritic recovery is possible in the hippocampal and pre-frontal cortex, this is only possible with the discontinuation of chronic stress (Conrad et al., 1999; Vyas et al., 2004). However, continuing dendritic retraction has a potential for damaging the hippocampus if persisting for months to years–referred to as the Glucocorticoid Vulnerability Hypothesis (Conrad, 2008).

Due to the various major stressors encountered by astronauts during LTSM, outer space presents an evolutionary mismatch with consequential risk for increasing neurotoxic challenges to emotional, learning and memory functions of the CNS. This is problematic since astronauts will be space-bound and isolated for a long duration when travelling to Mars.

## 4 Spaceflight-associated neuro-ocular syndrome (SANS)

From an ocular perspective the space environment presents critical challenges to human eyes and the visual system. In the absence of gravity, which has been a major evolutionary force in informing the structure, function and health of the human eyes, these organs are especially prone to visual pathologies such as spaceflight-associated neuro-ocular syndrome (SANS) (Mehare et al., 2024). This syndrome has been well documented in space medical literature given its relevance to neurological and mental health issues in astronauts. SANS presents as a collection of neuro-ocular symptoms due to a combination of factors such as exposure to micro-gravity, radiation, as well as, genetic factors (Ong et al., 2023; Mehare et al., 2024; Huang et al., 2019; Yang et al., 2022). Environmental increase in CO_2_, onboard lighting, high sodium intake and poor diet may also contribute to SANS (Mehare et al., 2024). However, microgravity plays a major role in the development of SANS, since micro-gravity may alter the distribution of CSF leading to increased cranial pressure (ICF), compression to the optic nerve and optic disc edema (Mehare et al., 2024; Mader et al., 2011). SANS may also induce intra/extraocular muscle atrophy with subsequent ocular impairments (i.e., blurred vision, hyperopic shift) (Paez et al., 2020). Importantly, there is a strong correlation between duration of flight and the development of SANS. Medical reports reveal that even short-term spaceflight (<2–3 weeks) may trigger visual disturbances (Paez et al., 2020), with increasing SANS related symptoms during LTSM (Mehare et al., 2024). Although, various potential treatments have been proposed (i.e., diuretics, nutritional supplements, anti-inflammatory drugs, neuroprotective agents, low vision aids) (Hartmann et al., 1977; Ghlichloo and Gerriets, 2023; Nucci et al., 2018) to mitigate SANS induced damage, astronauts will further need extensive monitoring and behavioural counselling due to the psychological impact of possible long-term visual alterations in space (Mehare et al., 2024). Moreover, more studies need to examine psychological stressors due to microgravity (Marsh, 2007).

## 5 Enteric gut microbiome and outer space: dysbiosis increases the risk of psychiatric diseases

The human intestinal tract, primarily the colon, is the site of the enteric gut microbiome (EGM) which consists of large communities of bacteria (numbering ∼ 10^14^) (Ley et al., 2006), comprising 150–400 species (Lloyd-Price et al., 2016), that differ significantly between individuals (The Human Microbiome Project Consortium, 2012), and which can alter over lifetime (Davenport et al., 2017; Odamaki et al., 2016; Aleman and Valenzano, 2019). The EGM is an important source of gene encoding as it contains a vast number of genes (>100 times greater than in the human genome) (O’Hara and Shanahan, 2006).

The EGM is involved in several key functions such as digestion, immune response (Shahab and Shahab, 2022), skeletal integrity, as well as neuro-behavioural and psychological functions (i.e., neuro-hormonal regulation, cognition (Davidson et al., 2018; Saniotis et al., 2020) and mood regulation (Desbonnet et al., 2008). Consequently, dysbiosis of the EGM can have a deleterious health outcome for individuals in several of these mentioned areas.

For millions of years hominins have co-evolved with microbial communities in the EGM. The EGM would have undergone continual evolutionary changes along with hominin adaptations to their environments and diets. For example, probably during the Pliocene/Pleistocene transition (∼3.2–2.6 Ma), the GIT of hominins underwent size reduction due to the introduction of meat and, later on, cooked food (Aiello and Wheeler, 1995; Henneberg et al., 1998; Mann, 2007). These two elements would have also contributed to alterations in the microbiome, which selected bacteria that favoured omnivorous diets (Shahab and Shahab, 2022).

Although, the human EGM is less diverse than that of the great apes, (Moeller et al., 2014; Davenport et al., 2017), intestinal biodiversity has further been altered by several bio-cultural changes from the Neolithic revolution (*circa* 10,000 ka) onwards. This trend was accelerated in the 20th century with global socio-cultural changes such as the introduction of anti-biotics, industrialised food products, increasing sedentism and chronic stress, and shifts to “western” style diets with significantly high omega-6 and inflammatory profiles (Bednarik et al., 2022), which have contributed to the “disappearing microbiome” (Blaser and Falkow, 2009).

Outer space represents a radical evolutionary mismatch for the EGM. It is well known that the EGM of astronauts is affected during outer space missions. Many stressors contribute to EGM dysbiosis during manned space missions which include microgravity, radiation, circadian desynchronisation cloistered living conditions and monotonous diets (Garrett-Bakelman et al., 2019; Voorhies et al., 2019; Marazziti et al., 2022; Arone et al., 2021; Siddiqui et al., 2021a; Siddiqui et al., 2021b; Mortazavi et al., 2024). The spacecraft/station environment does not contain many organisms that on Earth are sources of bacteria that may enrich the EGM. The EGM dysbiosis is significant since there are complex bidirectional interactions between the EGM and central nervous system (Kim et al., 2018; Saniotis et al., 2020), which also include microbiotic modulation of the vagus nerve *via* the microbiota-gut-vagus-brain-axis which may modify human behaviour (Montiel-Castro et al., 2013; Alcock et al., 2014). Interesting is that the probiotic influence of the EGM is annulled with either partial excision of the vagus nerve or vagotomy (Cryan and Dinan, 2012; Saniotis et al., 2020). Besides vagus transmitting sensations resulting from EGM’s activity (Liao et al., 2016) this may suggest that EGM produced neurometabolites are reliant on vagus nerve stimulation (Cryan and Dinan, 2012). Alterations to the microbiota-gut-brain-axis are associated with the pathogenesis of neurologic and psychiatric disorders (Cryan and Dinan, 2012; Kim et al., 2015; Mohajeri et al., 2018; Peterson, 2020).

Several types of microorganisms inhibit space-ship environments (Siddiqui et al., 2021a). Earlier microbiome studies found that astronauts were passing *Staphylococcus aureus* to each other (Pierson et al., 1996). A more recent International Space Ship (ISS) found that alteration to the EGM of astronauts increased inflammatory markers (*Fusicatenibacter* was negatively associated with “pro-inflammatory cytokines IL-8, IL-1b, IL4, and TNFa,” while genus *Dorea* was negatively correlated with cytokine IL-1b (Voorhies et al., 2019). It should be noted that studies have identified pro-inflammatory cytokines IL-8 and IL-1b as being implicated in higher risk of psychiatric pathologies (Tsai, 2021; Liu et al., 2022). In conclusion, the space environment increases the likelihood of EGM dysbiosis with subsequent risk of psychiatric disorders.

## 6 Circadian disruption during outer space missions and evolutionary mismatch

Like in other animals, human neurohormonal regulation is modulated by circadian rhythms. In humans, circadian rhythms regulate sleep/wake cycles, temperature, endocrine mechanisms, mental health, and wellbeing. Circadian synchronisation is an ancient adaptation to evolutionary environments in which the visceral neuroendocrine system (NS) was attuned to natural light fluctuations. In humans, the NS is regulated by a master biological clock (orchestrated by retinal, hypothalamic and reticular pathways), based on a 24 h cycle with light as the key zeitgeber (Anderson and Bradley, 2013). Any alteration of this circadian cycle (i.e., jetlag or shift workers) often leads to sleep disturbances or interruptions with subsequent reduced cognitive performance, tiredness, as well as higher risk for the onset of mental health problems (Anderson and Bradley, 2013).

Outer space represents a major mismatch for astronauts since they are removed from their evolutionary circadian environment. Astronautic sleep studies have identified that astronauts undergo sleep alteration due to circadian desychronisation, bright lighting, noisy internal spaceship environment, interior temperature, and sleeping bag discomfort (Arone et al., 2021; Marazziti et al., 2022). Even microgravity may diminish sleep duration (Gonfalone, 2016).

Analysis of sleep patterns of Shuttle crew members reported a 15% sleep reduction (Kelly et al., 2005), and 20 min less sleep during a 2-week mission relative to ground-based sleep (Barger et al., 2014). Consequently, due to insufficient additional light exposure in space, the 90-min light/dark cycle is disrupted eliciting homeostatic imbalance (Guo et al., 2014; Flynn-Evans et al., 2016; Wu et al., 2018). Despite NASA scheduling 8.5 h of sleep per night for crew members in space flight the average duration of sleep during space flight is just over 6 hours on Shuttle and ISS missions (Barger et al., 2014). Despite being allowed to sleep more hours astronauts still may have unproductive sleep quality (Flynn-Evans et al., 2016). Sleep alterations in astronauts are associated with increasing glucocorticoid secretion and inflammatory markers, memory and cognitive impairments, mood changes, altered relationship quality with crew members and reduced work performance (Chaumet et al., 2009).

Thus far, astronauts’ major recourse for improved sleep has been taking sleeping drugs and melatonin. However, long term use of benzodiazepine derivative sleeping medications (e.g., zolpidem midazolam, flurazepam, temazepam) that are regularly prescribed to astronauts are associated with various adverse side effects such as reduced cognitive and motor performance, drowsiness, *etc.* (Maust et al., 2019). Despite this, one study found that 71% of spaceship crew members were using sleep medications to promote or preserve sleep (Wotring, 2015).

## 7 “Earth-out-of-view-phenomenon”: neuro-behavioural risks of loss of connection with the natural world

*Homo* evolved in the natural world under intricate and on-going evolution. For tens of thousands of generations *Homo* evolved and adapted to various evolutionary environments which shaped its genome. The human body is an artefact of nature and exemplifies the complex gene/environment interplay. The entire emotional complex in *Homo*, both its neuro-hormonal substrates and psychosocial phenotypes were shaped and contoured by interaction with nature. In addition, the sensory perceptions which have been shaped by the natural world mediate our embodied awareness, which is intrinsically intertwined with multiple life forms “whether we are conscious of this connection or not” (Abram, 1996). The idea that natural environments promote human wellbeing has received considerable scientific attention over the last 3 decades. Many of these studies suggest that immersion in the natural world has neuro-behavioural and psychosocial benefits, such as reduced blood pressure and stress, shorter post-operative hospital period, lower mental fatigue and fewer sick days taken off work (Bowler et al., 2010; Velarde et al., 2007). Other cited psychological benefits from natural environments are promotion of calmness (Heerwagen, 2009), mental health improvement (Gullone, 2000), reduction in anger and aggression (Groenewegen et al., 2006) and enhanced natural killer (NK) immune response (Li et al., 2008). The theoretical frameworks of many of the afore-mentioned studies are informed mainly by E.O. Wilson’s “Biophilia Hypothesis” (2010), which argues that *Homo* has an innate preference for natural environments as a consequence of evolutionary history (Grinde and Patil, 2009).

The question remains that if psycho-somatic homeostasis is in some ways contingent on human/nature connection, what may be the mid to long term health consequences of astronauts stay in space? Will the evolutionary mismatch between the Gaian habitat and space lead to unknown health risks for future astronauts? Regarding physical health, some of these concerns have been addressed, and thus now regular exercise on board, recommendations for a balanced diet are common among astronauts, as well as sporadic leisure activities. However, much work still needs to be done on the psychological domain with long duration missions in perspective. On this note, some authors indicate that astronauts’ psychological status could be disrupted as the vision of planet Earth becomes increasingly difficult (Kanas and Manzey, 2008; Ihle et al., 2006). Moreover, astronauts from previous space missions stated that observing the Earth was meaningful and a positive factor of space travel (Kanas, 2011). Many interviewed astronauts and cosmonauts stated that being in space endowed them with a feeling of unity with nature, awe and a sense of transcendence–synonymous with “the overview effect” (Opsina et al., 2007). Since the so called “Earth-out-of-view-phenomenon” is new to the human psyche (Kanas and Manzey, 2008), it could well exacerbate feelings of homesickness, isolation and monotony (Kanas, 2011), with possible adverse mental health risks during LTSM.

## 8 Reduced natural selection in outer space and potential psychiatric disorders

Evolutionary medicine makes special reference to mechanisms operating within natural selection in ancestral hominins, and how alterations in selection processes are having health consequences in extant humans. *Homo* evolved on Earth and has adapted to the terrestrial environment. During most of hominin history, our ancestors were subjected to predation, parasitic and infectious diseases, trauma and environmental changes which shaped our genotypes (Saniotis and Henneberg, 2011b). Natural selection controlled the ratio between fitness enhancing/fitness compromising genes *via* differential reproductive success, which favoured traits that gave adaptive and reproductive advantages (Saniotis and Henneberg, 2011b). It is only with the advent of the Industrial Revolution of the 19th century that natural selection became relaxed due to improvements in public sanitation, nutrition and medicine (Saniotis and Henneberg, 2011b). Although this meant that most individuals in developed countries could reach reproductive adulthood, greater dependence on human interventions meant that natural selection had less ability to eradicate harmful alleles from the human gene pool. (Saniotis et al., 2020; Henneberg, 2025). It has been speculated that natural selection in the developed world is currently operating at only <1% compared with ∼50% prior to the Industrial Revolution (Saniotis and Henneberg, 2011b; Saniotis et al., 2020). Consequently, the proliferation of deleterious mutations may be leading to constrained fitness at a macro scale (Agrawal and Whitlock, 2012).

**FIGURE 1:**
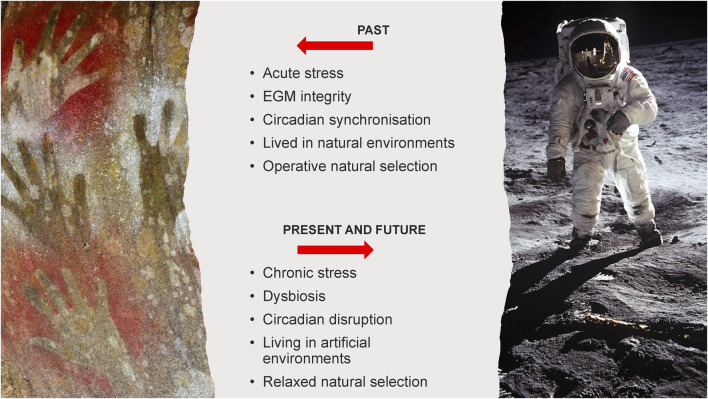
Types of evolutionary mismatch between past and extant/future humans.

In outer space, humans are not only removed from the natural environment where natural selection operates–an evolutionary mismatch, but are entirely dependent on human technology and medical interventions to sustain them (Saniotis et al., 2016). (Figure 1).

Given that there is a strong correlation between relaxed selection and mutation load, we can anticipate that this process may continue in humans during LTSM and in permanent space colonies on the moon and Mars. Additionally, long term radiation exposure could increase the incidence of deleterious mutations (Horneck et al., 2010).

On this theme, we must consider the possibility of potential alterations in gene expression regarding neuro-hormonal regulation in outer space. Popova et al. (2020) state that we have just begun to examine the association between risk neurogenes and behavioural disorders during LTSM. Limited animal studies during spaceflight identify that serotonergic and dopaminergic metabolic pathways, as well as neurotrophic factors (GDNF, CDNF) - key regulators of neuroplasticity are all altered (Popova et al., 2020; Rappaport and Corbally, 2023). Serotonin and dopamine are involved in emotion, motivation, memory, learning, sleep and motor skills. Neuroanatomical changes to the substantia nigra, hypothalamus and striatum have also been identified–brain areas involved in endocrine regulation, mood, cognition and movement are also vulnerable during spaceflight (Popova et al., 2020). These preliminary results indicate characteristics that correspond with Spaceflight Neuroplastic Syndrome (Rappaport and Corbally, 2023). It should be noted that the reported changes in rodent brain neuroanatomy and neurohormonal regulation occurred after only 1 month in space (Popova et al., 2020). Rappaport and Corbally (2023) argue that, although hundreds of astronauts seem to have been shielded from similar deleterious neurological deficits (possibly due to a higher level of neuroplasticity), this does not discount the effect of relaxed natural selection conferring greater probability for risk neurogenes, especially to future permanent space colonies.

## 9 Evolutionary based countermeasures for reducing psychopathologies during LTSM

**FIGURE 2:**
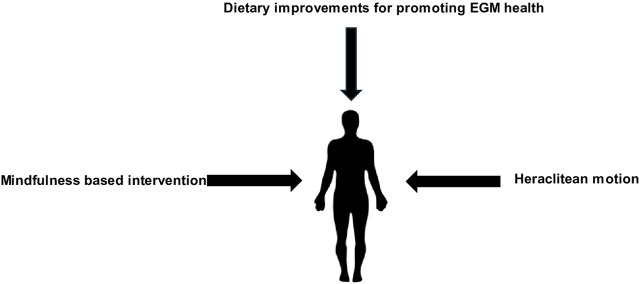
Evolutionary based countermeasures for reducing adverse mental health effects during LTSM.

For several decades, a plethora of space research has been accumulated regarding the physiological/psychological responses of astronauts during outer space missions. Thus far, scientists have been able to assess the health impact of astronauts, as well as the types of pre-mission acclimatising training necessary for alleviating exposure to the outer space environment (Criscuolo et al., 2020). Although humans have not adapted to living in outer space, it does not exclude that in the future, humans could be genetically engineered to better adapt to living in outer space for the long term. Human medical interventions have not only led to an unprecedented reduction in premature mortality since the 19th century, but are now preoccupied in developing techniques for artificially modifying the body at genetic and phenotypic levels (Saniotis and Henneberg, 2011b). We concur with Criscuolo et al. (2020) who point out that gene expression in outer space will have a major significance in the future evolution of permanent space colonists as we have suggested earlier regarding relaxation of natural selection. Due to psychiatric health issues that astronauts face, evolutionary medicine can provide various countermeasures for reducing the onset of psychopathologies and assisting astronauts’ mental health during LTSM. We anticipate that the following countermeasures may benefit astronauts since they are based on evolutionary antecedents (Figure 2).

### 9.1 Use of mindfulness based intervention for astronauts

Various authors have proposed the use of yoga and mindfulness based intervention (MBI) as a therapeutic countermeasure reducing psychological side effects of astronauts during space missions (Vernikos, 2012; Saniotis et al., 2023; Le Roy et al., 2023). These techniques have been used for millennia in various cultures and function in stimulating parasympathetic response while reducing sympathetic induced stress reaction. It has been argued that meditational techniques are based on evolutionary antecedents such as neuro-hormonally regulated auto-hypnotic response to threat, which had been positively selected due to its fitness value (McClenon, 1997; Krippner, 2000; Winkelman, 2000; 2004; Saniotis and Henneberg, 2011b). It has also been noted that Upper Palaeolithic shamans may have used altered states of consciousness in order to reduce individual ailments, improve fertility and increase group cohesion (Winkelman, 2000; 2004). Since relaxation inducing techniques may reduce an individual’s overall stress, this is important since astronauts have reported to have increasing stressors during LTSM (Marsh, 2007).

Mindfulness based intervention has been used in analog environments (Isolated and Confined Environments (ICE), Extreme and Unusual Environments (EUE)) with promising results in (i.e., better eating and sleeping habits, higher positive emotions states and lower negative emotions, reduced stress reactions) (Trousselard et al., 2010). Currently, more research needs to be conducted on whether MBI increases group cohesion among LTSM crew members or whether improving individual mindfulness has corresponding benefits at a group level (Le Roy et al., 2023). According to Saniotis et al. (2023), MBI is an inexpensive therapeutic technique that could be employed during pre-flight preparation and post-flight readaptation in order to assist astronauts’ mental health.

### 9.2 New strategies for addressing EGM in LTSM

Given that the EGM is altered during space conditions (i.e., radiation, microgravity, reduced biodiversity) it is difficult to develop new strategies in order to maintain its “normal” function. We encourage the further use of *Lactobacillus* and *Bifidobacterium* strains in probiotic form due to their anti-depressant mechanisms (Yong et al., 2020; Johnson et al., 2021; Dicks, 2022). Consequently, reduction of these species in the EGM have been linked to psychiatric disorders (Johnson et al., 2023). These bacteria can be freeze-dried and used, as has been the case of the *Lactobacillus casei* strain, which was used for 1 month by astronauts on the International Space Station (ISS) with positive results in improving intestinal microbial balance and innate immunity (Bharindwal et al., 2023). Future research needs to be conducted on developing probiotics that enhance mental health in astronauts, including bacterial strains not mentioned in this paper.

Second, we suggest the administration of faecal microbiota transplantation (FMT) to astronauts in order to recolonise beneficial gut bacteria where ordinary probiotic use is not efficacious due to the effects of antibiotic induced gut dysbiosis, microgravity, stress and radiation. Faecal microbiota transplantation provides a potential therapy in treating gut dysbiosis and restoring beneficial microbiota with subsequent mental wellbeing in astronauts during LTSM (Gupta et al., 2016). However, long term clinical trials need to be conducted on astronauts during space flight in order to verify the feasibility of FMT.

Third, we endorse the prioritising of omega-3 polyunsaturated fatty acids (PUFAs) in astronauts’ diets since they are critical to proper brain development and regulation. Human brain weight consists of approximately 50%–60% lipids, of which PUFAs account for 35% (Dighriri et al., 2022; Bos et al., 2016). Docosahexaenoic acid (DHA) is especially found in grey matter. It is well known that PUFAs are involved in numerous neuro-hormonal and cognitive processes (i.e., neurogenesis, neurotransmitter modulation, neuronal plasticity regulation, synthesis of neurotrophic factor BDNF) (Bednarik et al., 2022). Hominin intake of PUFAs increased during the Paleolithic period where the estimated ratio between omega-3 (α-linoleic acid) and omega-6 (linoleic acid) intake was ∼1:1 (Roccisano et al., 2016). In contrast, the ratio between omega-6/omega-3 in the western diet is ∼20:1. This difference is sufficient to increase the risk of psychiatric and neurological pathologies over time. For this reason, it is vital that astronauts maintain a healthy intake of PUFAs during LTSM.

### 9.3 Human space omics and personalised medicine

It is becoming clear that outer space has the potential to change gene expression which could affect metabolite synthesis and function across several neuro-physiological domains. As diverse human populations enter space natural selection will be further relaxed due to medical interventions and artificial conditions that will be necessary for humans to survive. We can only speculate to what extent gene-environment interaction may have in future gene expression of space travellers and colonists. For example, the enzyme Cytochrome P450 (CYP450), which is involved in ~ 75% of drug metabolism and is considerably polymorphic, has shown alterations in some astronaut gene profiles (Rutter et al., 2024). Additionally, a contemporary pharmacogenetics study found that 24 out of 78 standard drugs available to astronauts on the ISS were not effectively metabolised in polymorphic enzymes due to gene variants (Stingl et al., 2015; Rutter et al., 2024). These findings point to possible “omic derived molecular changes” in astronauts during prolonged space flight (Cope et al., 2022; Willis et al., 2021), Other studies have examined proteomic alterations in astronauts post LTSM (Brzhozovskiy et al., 2019; Pastushkova et al., 2021). Consequently, omic profiling has been proposed as a medical countermeasure in storing and using genetic data of astronauts (Cope et al., 2022). Such genetic data sets could lead to the development of personalised medicine which would facilitate metagenomic based health monitoring of astronauts in future LTSM (Danko et al., 2020).

### 9.4 Incorporating Heraclitean motion and attention restoration for promoting mental wellbeing in astronauts

In an earlier section, it was noted that humans have an inherent connection with nature as a result of their evolutionary history. It is an indisputable fact that human corporeality is rooted in sensuous engagement with the natural world, the latter operating as the inherent template for human biological and social processes (Saniotis et al., 2016). The loss of interaction with the natural world in outer space which has been referred to as “Earth-out-of-view-phenomenon” may have possible consequences in increasing psychopathologies especially during LTSM. We propose that developing relevant therapies to counter this problem should be based on the “Attention Restoration Hypothesis” which postulates that natural environments may provide temporary psychological relief from demanding cognitive routines that require high attention levels, such as various tasks performed by astronauts. Thus, short-term involvement in natural environments may reduce “depleted attentional resources” (Atchley et al., 2012). It has been identified that restoration occurs in the pre-frontal cortex, which regulates executive functions while also modulating hypothalamic-pituitary-adrenal axis (HPA) stress response (Winkelman, 2000; Saniotis et al., 2016).

Linked to attention restoration is the idea of “Heraclitean motion” which refers to environmental phenomena that are both static yet changing (i.e., fire, clouds, ocean, fish in an aquarium, fountains) (Katcher and Wilkins, 1993). According to some authors, the act of viewing phenomena that manifest Heraclitean motion produces a self-hypnotic state in the viewer after a few minutes (Saniotis et al., 2016). The relatively easy ability for humans to enter into self-hypnotic states was probably positively selected by natural selection since such states conferred ancestral hominins with reduced stress response to threat and induced relaxation which increased fertility and emotional wellbeing (McClenon, 1997; Saniotis and Henneberg, 2011a). Moreover, ancestral hominins may have gazed into fires which may have contributed to the evolution of cognitive abilities such as increased attention span, which is a hallmark of *Homo*. On this note, fire gazing was probably one of several behavioural and morphological characteristics which ancestral hominins were undergoing that were a result of self-amplifying feedback mechanisms probably starting already in the Miocene (Henneberg and Eckhardt, 2022; Henneberg, 2025). For example, the extant practice of fire gazing (*tatraka*) that has been practiced for centuries in India has been suggested to increase focus and attention, cognitive performance and mental relaxation (Kumar, 2010; Raghavendra and Singh, 2016; Chundawat and Panda, 2023).

We posit that effects of Heraclitean motion *via* prolonged exposure to the natural world may induce attention restoration. As Saniotis et al. (2016) note:

While more empirical studies are needed in testing the viability of Heraclitean motion in relation to astronauts, it seems likely that Heraclitean motion may improve individual resilience, especially in sensorially monotonous and enclosed environments such as spaceships.

1. We speculate that Heraclitean motion can be employed *via* the use of virtual reality (VR) technology that would provide images of natural environments to astronauts. Virtual reality technology could be employed at specified times during astronauts’work cycles as a way of restoring attentional resources, as well as countering the “Earth-out-of-view-phenomenon” in “home sick’ individuals. The use of VR as a countermeasure for the “Earth-out-of-view-phenomenon” has been identified by Gushin et al. (2021).
2. In the near future, holographic technology (HT) may also incorporate 3D natural environments in different parts of the space craft in order to restore attention resources and foster connection with the Earth. Thus far, holographic teleportation has been successfully employed in teleporting members that have been projected virtually on the International Space Station (Llaca and Davidson, 2023). It is anticipated that as HT improves, specific natural environments could be projected in various areas of spacecraft accompanied by sounds and smells of the respective nature-based holographs. The combination of visual, auditory and olfactory-based holographic natural environments would provide a more realistic experience of natural scenes, as well as necessary sensorial stimulation for countering the claustrophobic and tedious spaceship ambience. It is also feasible to suggest that holographs combining the sights, sounds and smells of nature could act as:
  1) an important sleeping aid for astronauts
  2) increase psychological wellbeing by reducing stress induced glucocorticoids and maintaining healthy neuro-hormonal regulation in astronauts.
3. Due to the significant musculoskeletal deterioration effects of microgravity, astronauts are required to exercise for approximately 2 hours per day using stationary bicycles or treadmills (Braddock, 2018). Although exercise fills a mandatory part in astronauts’ waking hours, this regime can be monotonous and boring. In the future, VR or HT can be employed during exercise programs where astronauts move through nature simulations (including sounds and smells of natural landscapes as well as flora and fauna). Such simulations would be designed to integrate executive, motor, sensory and limbic regions, thus providing cognitive, sensorial and emotional experiences for astronauts with subsequential neuroprotective and mental health benefits.

### 9.5 The problem of consciousness

Astronauts are highly educated and very intelligent people. Therefore, they may realise that their exposure to technologically created Earth environments is artificial. This realisation may interfere with them relaxing and restoring their attention. They may become stressed by what they will perceive as “fake” natural environments, especially that those may present them with repeated, like a broken record, experiences. During a space flight, there are real opportunities for observing Heraclitean motions–the slowly rotating view of the surrounding universe and of pondering details of the actual living environment that, although being a technological construct, is comprised of natural chemical compounds–plastics, metals, textiles, *etc.* Introducing astronauts to the acceptance of their immediate environment as a part of the nature may help in their personal adaptation to their unusual lifestyle. A spaceship consists of atoms linked in various ways to produce its structure. The ship moves through the space that contains the Earth and other objects. These objects move in regular ways that inform a rhythm of observing them. The schedule of human activities aboard a spaceship also has its rhythm, that, though artificial, is also informed by the biological requirements of human bodies–sleep, meals, exercise, relaxation, work. A spaceflight is a natural portion of the universal world where feedbacks between human needs and activities and their surroundings occur in ways somewhat different from the past evolutionary experience, but essentially the same systemic interactions among humans and their surroundings.

### 9.6 Peri anthropon: natural selection and humans in outer space

It is more than likely that human beings born in space stations over several generations will undergo changes in their genome resulting from altered mutation/selection balance. Some space environments may be mutagenic due to different from the Earth levels of radiation. Spontaneous mutations occur on Earth at high rates. The human germline mutation rate is approximately 1.2 × 10^−8^ per nucleotide per generation (Wood and Goriely, 2022).

Operation of natural selection in space stations/colonies will be strongly relaxed due to the availability of medical technologies and artificial living conditions. Thus, the accumulation of even mildly deleterious mutations over generations may lead to genetic load, which is evident in extant human populations (Saniotis et al., 2020; You and Henneberg, 2016; 2017; You et al., 2022). Allelic changes may occur faster in more mutagenic environments.

Although the operation of natural selection will be largely relaxed, some extreme effects of changed environments may affect the reproductive fitness of some colonists, thus leading to microevolutionary adaptations to particular space environments. Possible effects of inbreeding resulting from small effective population size, pressures of gravity, radiation and magnetic fields may trigger mutational variants causing neuro-neurobehavioural changes in ways that we do not yet fully understand.

Notwithstanding medical and dietary interventions in ensuring healthy gut microbiomes in astronauts and future space station colonists, relaxed natural selection will probably reduce microbiota variability with subsequent alterations in neuro-hormonal/neuro-trophic regulation and the growth of gut derived neurotransmitters. On this theme, Silva et al. (2023) note that changes to the gut microbiome will likely occur in response to the lack of exposure to widely varied microbiota colonies found in terrestrial environments (Silva et al., 2023).

As noted in numerous space studies, changes in gravity during space missions exert fundamental changes to astronauts’ bodies. The influence of gravity is significantly marked in growing offspring. Growth plates of human bones respond to mechanical forces acting on them (Alonso et al., 2022). Thus, children growing in an environment of lower force of gravity may have their growth disturbed in one way, e.g., growing very tall, while those growing under greater than Earth’s force of gravity will have growth disturbed in a different way, e.g., being very short and stocky. Concomitantly, gravity also affects muscle growth that is dependent on mechanical loads during their use. This, in turn contributes to proprioceptive sensory nerve signals from the musculoskeletal apparatus and motor signals to muscles which probably influence cognition. On this point, it has been speculated that motor neural circuits are implicated in thinking and cognition (Pilacinski et al., 2025; Baladron and Hamker, 2024). Therefore, voluntary muscular control, involuntary reflexes and cognitive abilities in inter-generational space station colonists could evolve differently from humans on Earth.

## 10 Conclusion

This paper has aimed to improve our understanding of the neuro-behavioural challenges which current astronauts face, as well as future astronauts and space colonists, from an evolutionary perspective. Evolutionary medicine has yet to be used as an explanatory model for addressing possible adverse mental health effects of humans during LTSM. This oversight needs to be corrected due to the increasing investment by space agencies and national governments in outer space exploration. Due to the evolutionary mismatches currently confronting astronauts’ mental health, this paper has discussed novel therapeutic countermeasures based on evolutionary principles for reducing adverse mental effects on astronauts and future space colonists. As we are still at a pioneering stage in space exploration the development of mental health strategies which privilege evolutionary principles will improve our current knowledge of bioastronautics.

## Data Availability

The original contributions presented in the study are included in the article/supplementary material, further inquiries can be directed to the corresponding author.
